# Pre-hospital ct diagnosis of subarachnoid hemorrhage

**DOI:** 10.1186/s13049-017-0365-1

**Published:** 2017-02-28

**Authors:** Maren Ranhoff Hov, Annette Ryen, Katrine Finsnes, Janne Storflor, Thomas Lindner, Jostein Gleditsch, Christian Georg Lund, on behalf of the NASPP Group

**Affiliations:** 1 The Norwegian Air Ambulance Foundation, Holterveien 24, 1448 Drøbak, Norway; 2 Department of Anaesthesiology, Østfold Hospital, Sarpsborg, Norway; 3 Department of Neurology, Østfold Hospital, Sarpsborg, Norway; 4 Department of Radiology, Østfold Hospital, Sarpsborg, Norway; 5 Department of Neurology, Oslo University Hospital, Rikshospitalet, Norway

**Keywords:** Pre-hospital, MSU, Cerebral CT, Subarachnoid hemorrhage, Stroke diagnostics, Neurosurgery

## Abstract

**Background:**

Subarachnoid hemorrhage (SAH) is associated with higher mortality in the acute phase than other stroke types. There is a particular risk of early and devastating re-bleeding. Patients therefore need urgent assessment in a neurosurgical department, and the shorter the time from symptom onset to diagnosis the better.

**Case presentation:**

The Norwegian Acute Stroke Pre-hospital Project (NASPP) has developed a Mobile Stroke Unit (MSU) model, which is staffed with anesthesiologists also trained in pre-hospital clinical assessment of acute stroke patients and interpretation of computerized tomography (CT). The MSU was operated on-call from the local dispatch center in a rural area 45–160 km away from a neurosurgical department. Two patients presented with clinical symptoms and signs compatible with SAH. In both cases, the CT examination confirmed the diagnosis of SAH. Both were transported directly from patient location to the regional neurosurgical department, saving at least 2–2.5 h of pre-neurosurgical time.

**Conclusion:**

The Norwegian MSU model staffed with anesthesiologists can rapidly establish an exact diagnosis of SAH, which in a rural area significantly reduces time to neurosurgical care.

**Trial registration:**

Study data are retrospectively registered in ClinicalTrail.gov. NCT03036020

**Unique Protocol ID:** NASPP-2

**Brief Title:** The Norwegian Acute Stroke Prehospital Project

**Overall Status:** Completed

**Primary Completion Date:** January 2016 [Actual]

**Verification Date:** January 2017

## Background

Subarachnoid haemorrhage (SAH) is a medical emergency, with overall incidence of approximately nine per 100.000 person-years [1]. SAH is associated with higher mortality in the acute phase than other stroke types, but immediate recognition and access to a neurosurgical department may reduce both mortality and morbidity [2]. A non-contrast CT scan will establish the diagnosis of SAH in most cases [3].

The Norwegian Acute Stroke Pre-hospital Project (NASPP) constructed a Mobile Stroke Unit (MSU) to explore the possibilities of pre-hospital diagnostics of acute stroke. The MSU was staffed with an anaesthesiologist from the national Helicopter Emergency Medical Service (HEMS), a paramedic and a nurse paramedic. The MSU anaesthesiologists were educated in making a clinical diagnosis of stroke, a National Institute of Stroke Scale (NIHSS) score, and a cerebral CT examination and interpretation [4]. The cerebral CT scan interpretation was primarily focused on identifying contraindications to thrombolysis for acute ischemic stroke, but also on the differentiation between types of intracranial haemorrhage.

## Methods

NASPP was initiated in October 2014 using the MSU (Mercedes Sprinter) with a cerebral CT scanner (CereTom Neurologica, Samsung), a point of care biochemical laboratory (pocH-100*i* Automated Hematology Analyzer and HEMOCHRON Jr. Signature±) and a telemedicine system (Meytech). The MSU’s catchment area was Østfold county, a rural and quite sparsely populated part of Norway with approximately 289 000 inhabitants (1720 inhabitants/sq. km). The distances to the only local acute hospital are up 100 km, and the distances to the neurosurgical department are between 45 and 160 km. The Emergency Medical Communication Centre (EMCC) for dispatch used the Norwegian index of emergency medicine as triage guideline [5]. The dispatch criteria included “acute thunderclap headache”.

We have included various time calculations in our study: estimated transit time from patient location to the local hospital, in hospital admission time and time for CT diagnostics, as well as time used for transfer from the local hospital to the neurosurgical department.

## Results

**Fig. 1:**
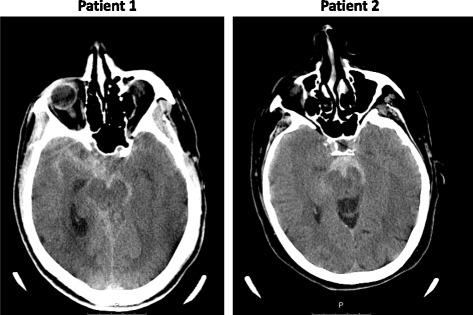
Pre-hospital cerebral CT scans from the MSU. PATIENT 1: Pre-hospital CT scan. There is subarachnoid hemorrhage located around the brainstem, in the supracellar cistern and in the right lateral fissure. PATIENT 2: Pre-hospital CT scan. There is subarachnoid hemorrhage mainly located ventral to the brainstem

During the NASPP study inclusion, the MSU performed 68 pre-hospital CT scans. In two cases (2.8%) the patient presented with hyper-acute headache as the main symptom, and subsequent CT scans showed intracranial bleeding compatible with SAH in both instances (Fig. 1).

The anaesthesiologist immediately interpreted the CT scan in the MSU and communicated the findings to the on-call radiologist at the local hospital. A joint decision was made to transport the patient directly to the neurosurgical department, and the on-call neurosurgeon was informed. Estimated pre-neurosurgical time saved by bypassing the local hospital was 120 and 150 min respectively. Full patient characteristics, time parameters and administered medications are shown in Table 1.

**Table 1:** Clinical patient characteristics, time parameters (min) and pre-hospital medication

	Patient 1	Patient 2
Age (years)	65	59
Sex	M	F
GCS (Glasgow Coma Scale)	15	14
Time from symptom onset to alarm MSU	4	171
Time from alarm MSU to MSU at the scene	25	33
Time from MSU at the scene to CT completed	30	30
Time from CT diagnostics to Neurosurgical Dept.	58	77
Time from symptom onset to neurosurgical department	127	312
Pre-hospital medication	Antiemetic	Tranexamic acid, analgesics, antiemetic
Age (years)	65	59
Sex	M	F
GCS (Glasgow Coma Scale)	15	14
Time from symptom onset to alarm MSU	4	171
Time from alarm MSU to MSU at the scene	25	33
Time from MSU at the scene to CT completed	30	30
Time from CT diagnostics to Neurosurgical Dept.	58	77
Time from symptom onset to neurosurgical department	127	312
Pre-hospital medication	Antiemetic	Tranexamic acid, analgesics, antiemetic

Both patients were treated at the Department of Neurosurgery at Oslo University Hospital. Patient 1 had a CT-angiography at arrival showing no signs of aneurysms. Due to thelarge volume of blood in the subarachnoid space a CT angiography was repeated, confirming the absence of vascular pathology. The patient subsequently developed signs of elevated intracranial pressure and was in need of external ventricular drainage. After 16 days the patient was discharged to the local hospital for further rehabilitation. Patient 2 also had a negative CT angiography and was conservatively treated at the neurosurgical ward for 4 days before discharge to home. Both patients are now living without any neurological dysfunction.

## Discussion

We have shown that the Norwegian MSU model is able to establish a definite pre-hospital diagnosis of SAH, which can substantially reduce pre-neurosurgical time. In spite of limited scientific evidence [2] there is reason to believe that SAH and its management is highly time-sensitive.

SAH is a subtype of stroke that generally affects a younger population. In SAH there is a significant risk of early and devastating re-bleeding, and hydrocephalus with subsequent increased intracranial pressure may develop acutely. Rapid transportation to a neurosurgical department is crucial to handle these complications [6]. In Norway, hardly any patient with acute headache is triaged to a neurosurgical department without an initial CTscan at the local hospital. If a similar study had been performed in the northern part of Norway, where distances to the local hospital can be more than 200 km, the time saved by establishing a pre-hospital SAH diagnosis could be several hours more than in our current study.

The main reason behind the development of the MSU concept has been to initiate thrombolytic treatment of acute ischemic stroke as early as possible after symptom start, preferably already in the “golden hour” [7]. Different MSU models for pre-hospital stroke diagnosis and treatment have been studied in recent years [8–11]. In the STEMO MSU study in Berlin, the delivery rate of patients with intracranial hemorrhage to hospitals without a neurosurgical department was 43.0% in conventional care and 11.3% in an MSU (*p* < 0.01) [12], allowing the authors to conclude that pre-hospital diagnostics might reduce time to neurosurgical care.

The different MSU-models in published literature are staffed either by a neurologist, a neurologist and a neuroradiologist, or solely by paramedics [8–10, 13]. Such MSU models may be very suitable in large urban areas, but the availability of specific trained individuals and in-hospital specialists makes it difficult to apply such MSU models to countries and regions that are less densely populated. In Norway, we have chosen to develop a MSU-concept similar to the national HEMS service in order to deliver stroke services to all parts of the country, rural and geographically very remote areas included. Moreover, when staffed with an anaesthesiologist a MSU may also provide resuscitation and medical emergency services such as endotracheal intubation and other invasive procedures to any unstable or critically ill patient.

Especially in rural areas, the concept of pre-hospital diagnostics and triage of acute cerebral stroke or head trauma may be as important for the MSU as providing early thrombolytic therapy. A positive cerebral radiological diagnosis, and nearly equally important a “negative finding”, will enable urgent pre-hospital medical decisions regarding to where and in which manner a patient with acute cerebral stroke or trauma should be transported. Such pre-hospital cerebral diagnostics can be carried out ambulatory by a MSU or possibly by a helicopter equipped with a cerebral CT-scanner in the future. Stationary CT scanners could by located for example at a district medical centre in rural areas. As demonstrated by our SAH patients, pre-hospital “acute brain” diagnostics will save a lot of time for the patients in a rural area, and probably increase quality adjusted life years (QUALYs).

## Conclusion

The Norwegian MSU model with anaesthesiologists can rapidly establish an exact diagnosis of SAH, which in a rural area can significantly reduce time to definitive neurosurgical care.
